# Coordinations between gene modules control the operation of plant amino acid metabolic networks

**DOI:** 10.1186/1752-0509-3-14

**Published:** 2009-01-26

**Authors:** Hadar Less, Gad Galili

**Affiliations:** 1 Department of Plant Sciences, the Weizmann Institute of Science, Rehovot 76100, Israel

## Abstract

**Background:**

Being sessile organisms, plants should adjust their metabolism to dynamic changes in their environment. Such adjustments need particular coordination in branched metabolic networks in which a given metabolite can be converted into multiple other metabolites via different enzymatic chains. In the present report, we developed a novel "Gene Coordination" bioinformatics approach and use it to elucidate adjustable transcriptional interactions of two branched amino acid metabolic networks in plants in response to environmental stresses, using publicly available microarray results.

**Results:**

Using our "Gene Coordination" approach, we have identified in Arabidopsis plants two oppositely regulated groups of "highly coordinated" genes within the branched Asp-family network of Arabidopsis plants, which metabolizes the amino acids Lys, Met, Thr, Ile and Gly, as well as a single group of "highly coordinated" genes within the branched aromatic amino acid metabolic network, which metabolizes the amino acids Trp, Phe and Tyr. These genes possess highly coordinated adjustable negative and positive expression responses to various stress cues, which apparently regulate adjustable metabolic shifts between competing branches of these networks. We also provide evidence implying that these highly coordinated genes are central to impose intra- and inter-network interactions between the Asp-family and aromatic amino acid metabolic networks as well as differential system interactions with other growth promoting and stress-associated genome-wide genes.

**Conclusion:**

Our novel Gene Coordination elucidates that branched amino acid metabolic networks in plants are regulated by specific groups of highly coordinated genes that possess adjustable intra-network, inter-network and genome-wide transcriptional interactions. We also hypothesize that such transcriptional interactions enable regulatory metabolic adjustments needed for adaptation to the stresses.

## Background

**Figure 1:**
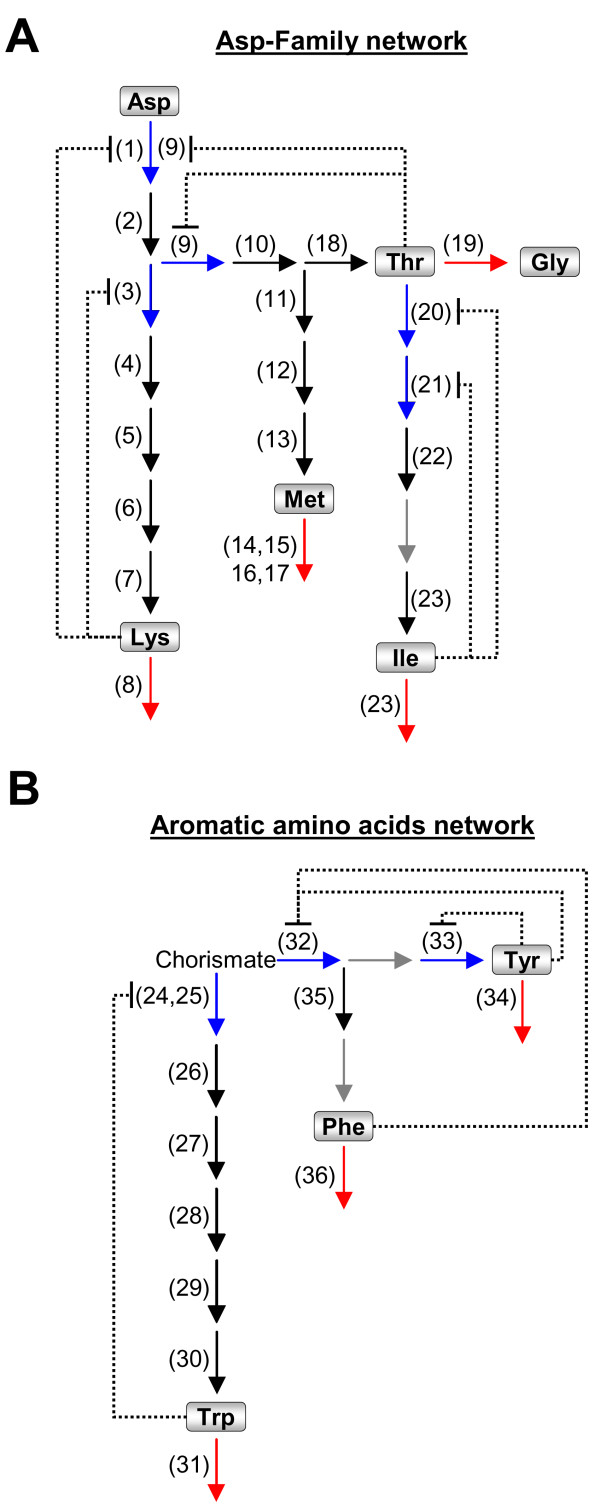
**Schematic representation of the Asp-family and aromatic amino acids metabolic networks analyzed in the present report**. The positions of the different amino acids in the different networks are marked in boxes. Biosynthetic/allosteric, biosynthetic/non-allosteric and catabolic enzymatic steps are indicated respectively by blue, black and red arrows, while enzymatic steps whose genes have not yet been identified are indicated by grey arrows. Numbers near each arrow refer to enzyme names as provided in Table 1. (A) The Asp-family network; (B) The Aromatic amino acids network. Dotted lines ending by a bar sign represent feedback inhibition loops.

Being stationary organisms that are unable to move, plants represent a unique biological system that is highly adaptive to environmental stresses. The adaptation mechanisms of plants to stresses also involve coordinated adjustments of a large array of metabolic networks. Among those are metabolic networks containing amino acids as intermediate metabolites, which can either be incorporated into proteins, accumulate to high levels in response to specific cues, such as proline accumulation in response to salt stress [1], or serve as precursors for a large array of metabolites with multiple functions. Some of the metabolic networks of amino acids possess several competing branches, each leading to the synthesis of one or more amino acids and their downstream metabolites. Two of the most important branched metabolic networks of amino acids are the Asp-family network and the aromatic amino acids (AAA) network (Fig. 1 panels A and B). These two networks also possess significant nutritional value because humans and much of their farm animals cannot synthesize the amino acids produced by them and therefore depend on plants as their nutritional supplements [2].

**Table 1:** A list and additional relevant information of all genes belonging to the Asp-family and the aromatic amino acids (AAA) networks analyzed in this report.

**Pathway**	**Enzyme**	**Step^a^**	**Symbole**	**ATG^b^**	**Probeset^c^**
Lys met.	monofunctional aspartate kinase	1	AK^d^	AT3G02020	258977_s_at
			AK^d^	AT5G14060	258977_s_at
			AK1	AT5G13280	250291_at
	aspartate-semialdehyde dehydrogenase	2	ASD	AT1G14810	262841_at
	dihydrodipicolinate synthase	3	DHDPS1	AT3G60880	251392_at
			DHDPS2	AT2G45440	245145_at
	dihydrodipicolinate reductase	4	DHDPR	AT2G44040	267237_s_at
			DHDPR	AT3G59890	267237_s_at
			DHDPR	AT5G52100	248402_at
	L, L-diaminopimelate aminotransferase	5	AGD2	AT4G33680	253308_at
	diaminopimelate epimerase	6	DAPE	AT3G53580	251948_at
	diaminopimelate decarboxylase	7	DAPD^d^	AT3G14390	258365_s_at
			DAPD^d^	AT5G11880	258365_s_at
	lysine-ketoglutarate reductase/saccharopine dehydrogenase	8	LKR/SDH^d^	AT4G33150	253373_at
Met met.	aspartate kinase/homoserine dehydrogenase	9	AK/HSDH1^d^	AT1G31230	263696_at
			AK/HSDH2	AT4G19710	254535_at
	aspartate-semialdehyde dehydrogenase	2	ASD	AT1G14810	262841_at
	homoserine kinase	10	HSK	AT2G17265	264855_at
	cystathionine γ synthase	11	CGS1^d^	AT3G01120	259279_at
			CGS	AT1G33320	256531_at
	cystathionine β lyase	12	CBL	AT3G57050	251666_at
	methionine synthase	13	MS1	AT5G17920	259343_s_at
			MS2	AT3G03780	259343_s_at
			MS3	AT5G20980	246185_at
	homocysteine S-methyltransferase	14	HMT1	AT3G25900	258075_at
			HMT2	AT3G63250	251175_at
			HMT3	AT3G22740	258322_at
	S-adenosylmethionine synthetase	15	SAMS1	AT1G02500	260913_at
			SAMS2	AT4G01850	255552_at
			SAMS3^d^	AT3G17390	258415_at
			SAMS4	AT2G36880	263838_at
	methionine γ lyase	16	MGL^d^	AT1G64660	261957_at
	methylthioalkylmalate synthase	17	MAM1^d^	AT5G23010	249866_at
			MAML^d^	AT5G23020	249867_at
	methionine-oxo-acid transaminase		BCAT4^d^	AT3G19710	257021_at
Thr met.	aspartate kinase/homoserine dehydrogenase	9	AK/HSDH1	AT1G31230	263696_at
			AK/HSDH2	AT4G19710	254535_at
	aspartate-semialdehyde dehydrogenase	2	ASD	AT1G14810	262841_at
	homoserine kinase	10	HSK	AT2G17265	264855_at
	threonine synthase	18	TS	AT1G72810	262380_at
			TS	AT4G29840	253700_at
	threonine aldolase	19	THA1^d^	AT1G08630	264777_at
			THA2	AT3G04520	258599_at
Ile met.	threonine deaminase	20	TD	AT3G10050	258884_at
	acetolactate synthase	21	AHASS1	AT2G31810	263460_at
			AHASS2	AT5G16290	250111_at
			AHAS	AT3G48560	252325_at
	ketol-acid reductoisomerase	22	KARI	AT3G58610	251536_at
	branched-chain aminoacid aminotransferase	23	BCAT1	AT1G10060	264525_at
			BCAT2^d^	AT1G10070	264524_at
			BCAT3^d^	AT3G49680	252274_at
			BCAT5	AT5G65780	247158_at
			BCAT6	AT1G50110	261636_at
			BCAT7	AT1G50090	261690_at
Trp met.	anthranilate synthase β	24	ASB^d^	AT1G24807	247864_s_at
			ASB^d^	AT1G24909	247864_s_at
			ASB^d^	AT1G25083	247864_s_at
			ASB^d^	AT1G25155	247864_s_at
			ASB^d^	AT1G25220	247864_s_at
			ASB^d^	AT5G57890	247864_s_at
	anthranilate synthase a	25	ASA1^d^	AT5G05730	250738_at
			ASA2	AT2G29690	266671_at
			ASA	AT3G55870	251716_at
	anthranilate phosphoribosyltransferase	26	TRP	AT1G70570	260311_at
			TRP1	AT5G17990	250014_at
	phosphoribosylanthranilate isomerase	27	PAI1	AT1G07780	259770_s_at
			PAI2	AT5G05590	259770_s_at
			PAI3	AT1G29410	259770_s_at
	indole-3-glycerol phosphate synthase	28	IGPS^d^	AT2G04400	263807_at
			IGPS	AT5G48220	248688_at
	tryptophan synthase a	29	TSA2^d^	AT3G54640	251847_at
			TSA	AT4G02610	255487_at
	tryptophan synthase β	30	TSB1^d^	AT5G54810	253898_s_at
			TSB2^d^	AT4G27070	253898_s_at
			TSB	AT5G38530	249515_at
	cytochrome P450	31	CYP79B3	AT2G22330	264052_at
			CYP79B2^d^	AT4G39950	252827_at
Phe & Tyr met.	chorismate mutase	32	CM1	AT3G29200	257746_at
			CM2	AT5G10870	250407_at
			CM3	AT1G69370	260360_at
	arogenate dehydrogenase	33	AAT1	AT5G34930	255859_at
			AAT2	AT1G15710	259486_at
	tyrosine aminotransferase	34	TAT3^d^	AT2G24850	263539_at
			TAT	AT5G53970	248207_at
	prephenate dehydratase	35	PD1	AT2G27820	266257_at
			PD	AT1G08250	261758_at
			PD	AT1G11790	262825_at
			PD	AT3G07630	259254_at
			PD^d^	AT3G44720	252652_at
			PD	AT5G22630	249910_at
	phenylalanine ammonia-lyase	36	PAL1^d^	AT2G37040	263845_at
			PAL2^d^	AT3G53260	251984_at
			PAL3	AT5G04230	245690_at

^a^Step numbers represent enzymatic steps described in Fig. 1 and Additional file 1.

^b^ATG represents the common locus number using TAIR nomenclature.

^c^Probeset represents specific identifiers according to Affymetrix AtH1 microarray.

^d^Highly Coordinated genes (HCGs)

The Asp-family network, which classically includes the amino acids Lys, Thr, Met and Ile (Fig. 1 panel A), is a central regulator of plant growth not only because its amino acids are essential for protein synthesis. Met is a metabolic precursor for multiple fundamental cellular processes [3-8], while Thr, through its conversion into Ile, participates plant pathogen interactions [9,10] and cellular energy production [11]. Thr is also catabolized by two Thr aldolase isozymes into Gly, which is closely associated with photorespiration [12,13]. Lys biosynthesis and catabolism was also shown to be associated with plant pathogen interactions as well as with the production of the stress associated hormone salicylic acid [14,15]. The AAA metabolic network leads to the synthesis of Trp, Phe and Tyr, which are further used as precursors for various hormones, cell wall components and a large array of multifunctional secondary metabolites [8,16-18].

Using a bioinformatics approach based on publicly available microarray results, we have recently demonstrated evidence supporting the presence of principal transcriptional programs of amino acid metabolism in response to abiotic stresses [19]. Strikingly, these responses were most profoundly associated with changes in expression of genes encoding the catabolic enzymes of the amino acids with only minor changes in mRNA levels of genes encoding the biosynthesis enzymes [19]. This phenomenon in which genes encoding only a fraction of the enzymes within a given metabolic pathway are altered in response to a given cue is in sharp contrast to microorganisms in which the expression of all genes of a given metabolic pathway is generally altered in response to a given cue [20]. This major conceptual difference is likely because highly dividing microorganisms face extensive dilution of their enzyme concentrations through the frequent cell divisions, while cells of higher organisms relatively infrequently divide in the short time scales of environmental changes. In addition, higher organisms often have more than one gene encoding a given enzyme, and therefore transcriptionally regulate only a fraction of the genes encoding isozymes in different tissues or in response to different cues. This fact adds another damnation of complexity when analyzing transcription results of higher organisms. Notably, Arabidopsis plants specifically adjust the expression of genes encoding different catabolic enzymes of amino acids in response to different stresses [19]. This implies that bioinformatics approach aiming to elucidate interactions of different branched networks of amino acid metabolism in plants under multiple growth conditions is a challenging issue because different branches are likely subjected to dynamically changing patterns of coordinated regulation. Under some conditions, such as optimal growth conditions, all branches are expected to operate efficiently to allow the synthesis of amino acid for incorporation into proteins. Under other biological perturbations, such as exposure to specific stress conditions, fluxes are expected to increase in some branches on the expense of others to allow optimal metabolic adjustments [19]. Therefore genes encoding enzymes of specific branches within a given amino acid metabolic network are expected to be both positively correlated under some biological perturbations, while negatively correlated or not correlated at all under other biological perturbations. As a consequence, genes encoding enzymes of specific branches within a given metabolic network are also expected to be both positively correlated, negatively correlated or non-correlated with other genome-wide genes under different biological perturbations. Therefore, commonly used correlation methodologies are not suited to elucidate transcriptional network interactions of amino acid metabolic pathways because they are unable to resolve positive from negative correlations within a wide range of biological perturbations. To overcome this limitation, we have developed in the present report a novel principal approach, termed "gene coordination" allowing the elucidation of groups of genes whose expression is coordinated by both positive and negative correlations in response to different sets of biological perturbations. We also used this novel approach to elucidate differential genome-wide interactions of two central amino acid metabolic networks, namely the Asp-family and AAA metabolic networks,. Our results expose novel principal transcriptional regulatory aspects of each of these networks and also show that they differentially interact with genome-wide genes in plant growth and response to the environment.

## Results

### Selection of genes associated with the Asp-family and AAA metabolic networks and sources of publicly available microarray experiments

The aim of this research was to discover novel network interactions associated with two central metabolic networks of amino acids, namely the Asp-family network and the AAA network (Fig. 1 panels A and B). The Asp-family network includes the amino acids Lys, Thr and Met, Ile and Gly whose synthesis initiates from Asp, and in which one pathway leads to Lys metabolism (Fig. 1, enzymatic steps 1–8), a second pathway leads to Thr synthesis and its further conversion into Ile and Gly (Fig. 1, enzymatic steps, 2, 9, 10, 18–23), and the third pathway leads to Met metabolism (Fig. 1, enzymatic steps 2, 9–17). The AAA network, whose synthesis initiates from chorismate, includes three pathways; one leading to Trp metabolism (Fig. 1, enzymatic steps 24–31), while the second and third pathways lead to Phe and Tyr metabolism. Since the Phe and Tyr pathways contain altogether only few enzymatic steps (Fig. 1, enzymes 32–36), from which one is common to both pathways, we therefore treated them as a single pathway. In both the Asp-family and AAA networks, we focused on genes encoding biosynthetic enzymes as well as enzymes catalyzing the first catabolic steps of the amino acids. Our definition of catabolism included the breakdown of the amino acid into carbon, nitrogen and energy-associated molecules as well as the utilization of the amino acids for the synthesis of other special metabolites, such as secondary metabolites. The selection of the entire set of genes associated with these two networks was as previously described [19]. The functional annotation of the genes in this list was based on a combinatorial analysis of information form TAIR , ARACYC  and literature review. The list of enzymes and the genes encoding them, which were used in this study, are detailed in Table 1. Some of the genes studied in this report are indistinguishable in the ATH1 Affymetrix chip and are monitored by a common probeset as indicated in Table 1. For simplicity, we will refer in the following to these probesets as genes rather than probesets.

Our bioinformatics approach was based on analyzing a selected set of experiments in the publicly available Arabidopsis microarray data available through the NASC database . Yet, since we were interested to elucidate programs of transcriptional regulation, we focused only on relatively short time responses (mostly up to 24 h) of genes, belonging to the Asp-family and AAA metabolic networks, to different biological cues. In addition, in experiments where a mutant genotype was analyzed in comparison to a control genotype, we considered these lines as two distinct genotypes. These guidelines enabled us to extract 211 different short-term biological perturbations, which were enforced on many different genotypes. It is important to emphasize that in cases in which multiple time points were analyzed in an individual experiment, each time point was used as an independent biological perturbation. This was done due to the fact that the response of different genes in different time scales (temporal gene coordination) may play a significant regulatory role in the adjustment of metabolic channelling as was suggested in our previous report [19].

Since different branches in branched metabolic networks lead to the synthesis of different metabolites with distinct functions, it is expected that the different branches should possess significant regulatory gene expression flexibilities to either be positively or negatively correlated in response to different cues (see Introduction section). Hence, Pearson correlation analysis is not suitable to discover associations between such genes over large sets of different experiments. To overcome this, we developed a novel bioinformatics approach, termed "Gene Coordination". The principal difference between gene correlation (such as Pearson correlation) and gene coordination is that gene coordination allows the identification of both statistically significant negative and positive coordinations of expression between pairs of genes over a set of multiple biological perturbations. In short, this novel measure assigns two distinct values between each pair of genes: (i) a positive coordination, which is the number of biological perturbations in which both genes of a given gene pair are either up-regulated or down-regulated together in a statistical significant manner, compared to non-treated controls; and (ii) a negative coordination which is the number of biological perturbations in which one gene of the same gene pair is up-regulated while the other is down-regulated in a statistical significant manner, compared to non-treated controls. In contrast to Pearson correlation, which is usually calculated using the absolute value of each hybridization experiment and when calculated based on expression differences ignores the statistical significance of the differences in each biological perturbation, the gene coordination measure is always based on expression differences and the statistical significance of the difference in each biological perturbation is always taken into account. It is important to mention that since the calculation of gene coordination is strongly based on the selection of the different biological perturbations, it is essential to identify the relevant biological perturbations for each biological question. Detailed explanation of this novel measure and the statistical model that was used to analyze it are described in the Methods section.

**Figure 2:**
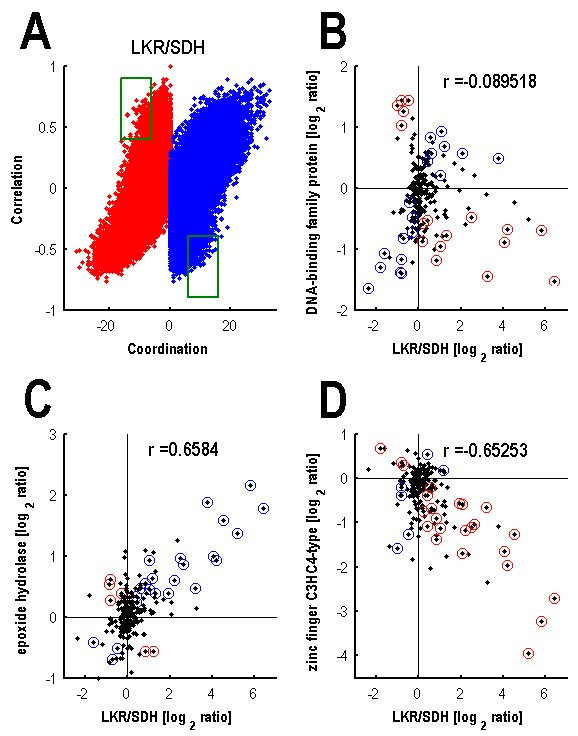
**Illustration of the gene coordination principal, using the LKR/SDH gene as an example**. (A) Relationship between Pearson correlation and gene coordination values, exemplified for the LKR/SDH gene probed against the entire genome-wide set of Arabidopsis genes. Each blue dot represents the relationship between Pearson correlation (Y axis) and positive coordination (X axis), while each red dot represents the relationship between Pearson correlation (Y axis) and negative coordination (X axis), calculated across the entire set of 211 biological perturbations. The two green rectangles indicate areas containing genes pairs having high positive correlation and a significant negative coordination across the entire of biological perturbations (upper left rectangle) and vice versa (lower right rectangle). (B, C, D) Relationships between the expression differences of the LKR/SDH gene and three other representative genes across the entire set of 211 biological perturbations. Each black dot indicates the expression difference (treatment versus control) in response to a single specific biological perturbation. Black dots inside red circles indicate perturbations that contribute to a negative coordination, while black dots inside blue circles indicate perturbations that contribute to a positive coordination between each of the two compared genes. The Pearson correlation value is indicated on the top of each panel.

The power of our novel gene coordination measure is exemplified in Fig. 2. Panel A illustrates the relationships between the correlation and coordination of expression of the LKR/SDH gene of lysine catabolism with each one of the other Arabidopsis genes (monitored by the ATH1 Affymetrix microarray), based on our entire dataset of 211 different biological perturbations. This relationship is depicted by two dotes, one with a blue color and the second with a red color. The two dotes possess identical correlation values over the entire dataset of 211 biological perturbations (Y axis) because they belong to the same pair of genes. Yet, they possess two different coordination values, one showing negative coordination, which is contributed by a selected set of biological perturbations (a red spot with a negative value on the X axis), while the other showing positive coordination that is contributed by a different set of biological perturbations (a blue spot with a positive value on the X axis) (see Methods section for details). Panels B-D of Fig. 2 illustrate the expression relationship between the LKR/SDH gene and three representative genes over the entire dataset of 211 biological perturbations. Each dot represents data associated with a specific biological perturbation that was obtained from analysis of at least four different microarray chips and thus allows performing a significance test. R value of the Pearson correlation coefficient is also indicated within each panel. Panel B illustrates a case in which there is no correlation between the pair of genes across the entire depicted set of biological perturbations, but there is a significant number of biological perturbations that contribute both to negative and positive coordinations between these two genes (red and blue circled dots, respectively). Panel C illustrates a case in which there is a positive correlation of expression between a pair of genes across the entire depicted set of biological perturbations, but a significant number of biological perturbations contribute to a negative coordination between these two genes (red circled dots). Panel D illustrates an opposite case in which there is a negative correlation of expression between a pair of genes across the entire depicted set of biological perturbations, but a significant number of biological perturbations contribute to a positive coordination between these two genes (blue circled dots). Hence, genes whose expression shows statistically significant negative or positive coordination under depicted set of biological perturbations (derived from the 211 short-term biological perturbations drawn from the NASC database) were defined as "highly coordinated genes" (HCGs).

### Identification of highly coordinated genes (HCGs) within the Asp-family and AAA networks

**Figure 3:**
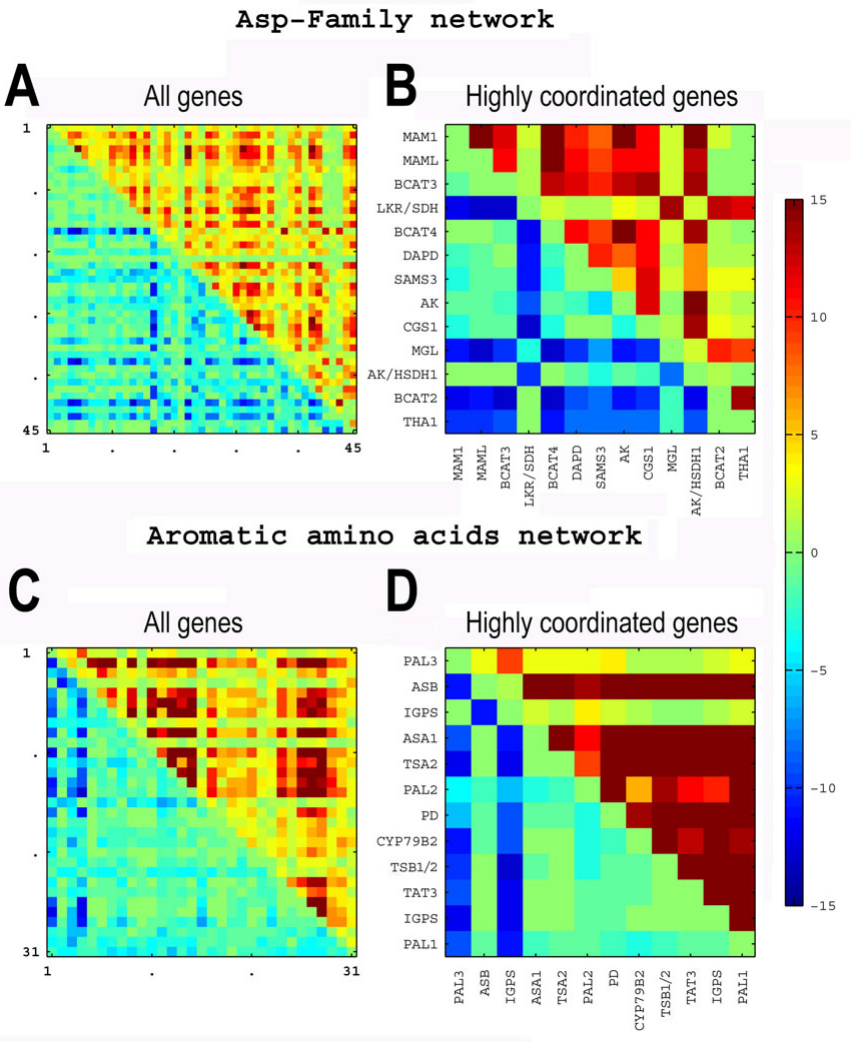
**Gene coordination matrixes of the Asp-family and aromatic amino acid metabolic networks**. Coordination matrixes calculated for the entire set of genes belonging to the Asp-family network (panel A) and the Aromatic amino acids (AAA) network (panel C) and only for the highly coordinated genes (HCGs) of each network (panels B and D, respectively). In each coordination matrix, the upper right triangle represents positive coordination while the lower left triangle represent negative coordination, obtained under different biological perturbations. The numbers in panels A & C represent the entire set of genes in each network. The color scale for all matrixes is indicated on the right.

To identify the HCGs within each network, we used a coordination matrix (using heat map representation) in which the upper right triangle shows positive coordination and the lower left triangle shows negative coordination (Fig. 3). It is important to note that in such a coordination matrix, the red or the blue squares existing for each genes pair are derived from responses to different biological perturbations. Panels A and C in Fig. 3 depict respectively the coordination matrixes of the entire set of genes within the Asp-family and AAA metabolic networks. Notably, only a selected group of genes within each of these two networks showed either high negative (blue squares) and/or high positive (red squares) coordination of expression in which higher intensity of the colors signifies higher number of biological perturbations in which negative or positive co-regulation were observed. In order to identify the HCGs within each network, we used a background model for the evaluation of the negative and positive coordination in each network, assuming no coordination, and considered genes to be HCGs if their observed coordination was higher by more then six standard deviations from the coordination that was estimated by our background model (for detail see Methods section). Based on these criteria, we selected 13 and 12 HCGs within the Asp-family and the AAA networks, respectively (Fig. 3, respective panels B and D). It is clear from these two panels that in each network, the HCGs are divided into two distinct groups of genes in which there is a positive coordination between the genes within each HCGs group and a negative coordination between the genes of the different HCGs groups. In the Asp-family network, one HCGs group includes exclusively catabolic genes, namely THA1 (Thr catabolism into Gly), BCAT2 (Ile metabolism), MGL (Met catabolism into methanethiol as well as to 2-oxobutyrate on route to Ile production) and LKR/SDH (Lys catabolism into Glu and aspartic semialdehyde) (see Table 1 and Additional file 1), and from here on will be referred to as the "Catabolic group". The second group in the Asp-family network includes almost entirely genes encoding enzymes of Met biosynthesis and catabolism towards SAM and various glucosinolates, and therefore from here on will be referred to as the "Met metabolism group". In specific, this group includes (i) genes encoding biosynthetic enzymes, namely, the bifunctional AK/HSDH1 isozyme plus one or more monofunctional AK isozymes catalyzing the first step of the Asp-family network (AT3G02020 and AT5G14060 genes whose highly homologous DNA sequences are undistinguishable using the Affymetrix ATH1 chip), CGS1 (Met biosynthesis) and DAPD (Lys biosynthesis); and (ii) genes encoding catabolic enzymes, namely, SAMS3 (Met catabolism into SAM), BCAT3 (Ile metabolism) as well as BCAT4, MAM1 and MAML (Met catabolism towards biosynthesis of glucosinolates) (see additional file 1).

In the AAA network, one HCGs group includes 10 genes containing both biosynthetic and catabolic genes, while the second HCGs group includes only two genes (PAL3 and IGPS). Since the expression patterns of the AAA genes showed that the first group also possesses isozymic genes of PAL and IGPS, which are expressed under conditions that PAL3 and IGPS are repressed (data not shown), we decided to exclude this group from further analyses. The first group, which will be referred to from here on as the sole "AAA group" of the AAA network, includes the following genes: The Trp biosynthesis genes ASA1, ASB, TSA2, TSB1/2 and one of the IGPS genes; the Trp catabolic gene CYP79B2, the Phe biosynthesis gene PD, the Phe catabolic genes PAL1 and PAL2 and the Tyr catabolic gene TAT3 (see Table 1 and Additional file 1).

### Effects of specific biological perturbations on the patterns of expression of the HCGs of the Asp-family network

**Figure 4:**
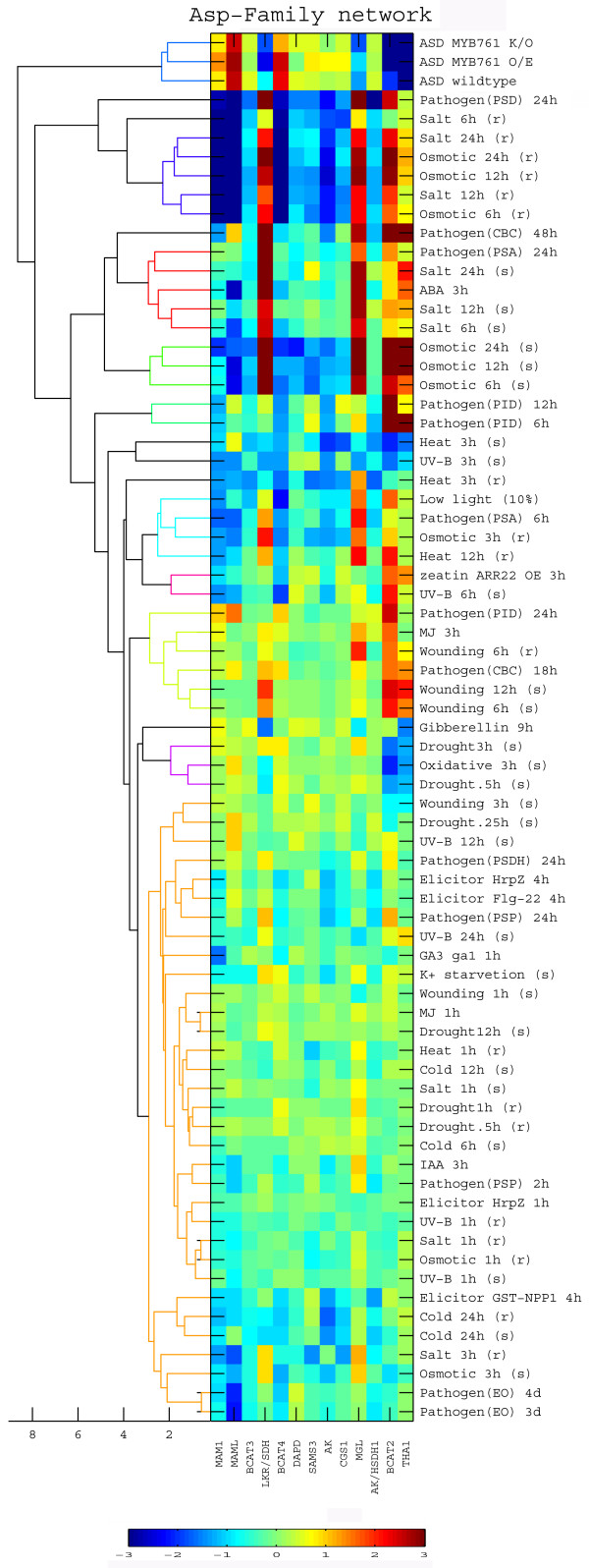
**Identification of highly coordinated genes within the Asp-family and aromatic amino acid metabolic networks**. Clustering of the entire set of biological perturbations having a significant effect on at least one of the highly coordinated genes (HCGs) of either the Asp-family or the aromatic amino acids networks based on their effects on the expression of the Asp-family network HCGs. The dendrogram on the left shows the Euclidian distance tree, while the heat map in the center shows the response of the different HCGs to the different biological perturbations that are indicated on the right. The color scale bar is indicated on the bottom. Abbreviation of specific biological perturbations: ASD, adding sucrose in the dark; (s), shoot; (r), root; CBC, Conidiospores of *Botrytis cinerea*; EO, *Erysiphe orontii*; PID, *Phytophthora infestans *drops; PSA, *Pseudomonas syringae *pv tomato avrRpm1; PSD, *Pseudomonas syringae *pv tomato DC3000; PSDH, *Pseudomonas syringae *pv tomato DC3000 hrcC-; PSP, *Pseudomonas syringae *pv phaseolicola.

To identify the patterns of response of the HCGs of the Asp-family network to specific biological perturbations, we selected from the 211 short-term biological perturbations of the NASC database all biological perturbations in which expression of at least one of the HCGs of this network was increased or decreased by more then four folds. This stringent filter was used to make sure that we are analyzing only relevant perturbations. In experiments containing time course exposure to a given cue, each time point was considered separately. Fig. 4 lists the specific biological perturbations obeying the above rule (right part), as well as their effect on the expression of each of the HCGs of the Asp-family network (central matrix) and the Euclidian distance between the specific biological perturbations (dendogram on the left). In this dendogram, the larger the separation between two biological perturbations, the larger is the extent of their differential effects on the HCGs of the Asp-family network. In addition, an artificial biological perturbation of growing in the dark in the presence of sucrose also had a significant effect on the Asp-family network (Fig. 4, the top three biological perturbations associated with MYB761). The magnitudes of the expression response of the different HCGs to the different abiotic and biotic stresses varied between the different stresses and the time course of each stress, being generally higher at later periods of exposure to the stresses (Fig. 4). However, the HCGs could be clearly classified into two principal groups, matching the earlier "Met metabolism" and "Catabolic" HCGs groups with the opposite responses, the first (Met metabolism group) being repressed (Fig. 4; squares with varying blue color), while the second (catabolic group) being induced (Fig. 4: squares with yellow and red colors) by the stresses.

Notably, the artificial biological perturbation of dark plus added sucrose on plants with suppression and over expression of MYB761 (Fig. 4, top three biological perturbations) had a significantly different effect on the Asp-family genes repressing the expression of the genes belonging to the catabolic group (THA1, BCAT2, MGL and LKR/SDH), while inducing the expression of genes belonging to the Met metabolism group (MAM1, MAML, BCAT4 as well as to a lower extent also CGS, AK and SAMS3). This artificial biological perturbation seems to increase the biosynthetic fluxes and reduce much of the catabolic fluxes of the Asp-family network and may signify an override of the sucrose signal over the dark signal to promote growth and protein synthesis.

### Effects of specific biological perturbations on the patterns of expression of the HCGs of the AAA network

**Figure 5:**
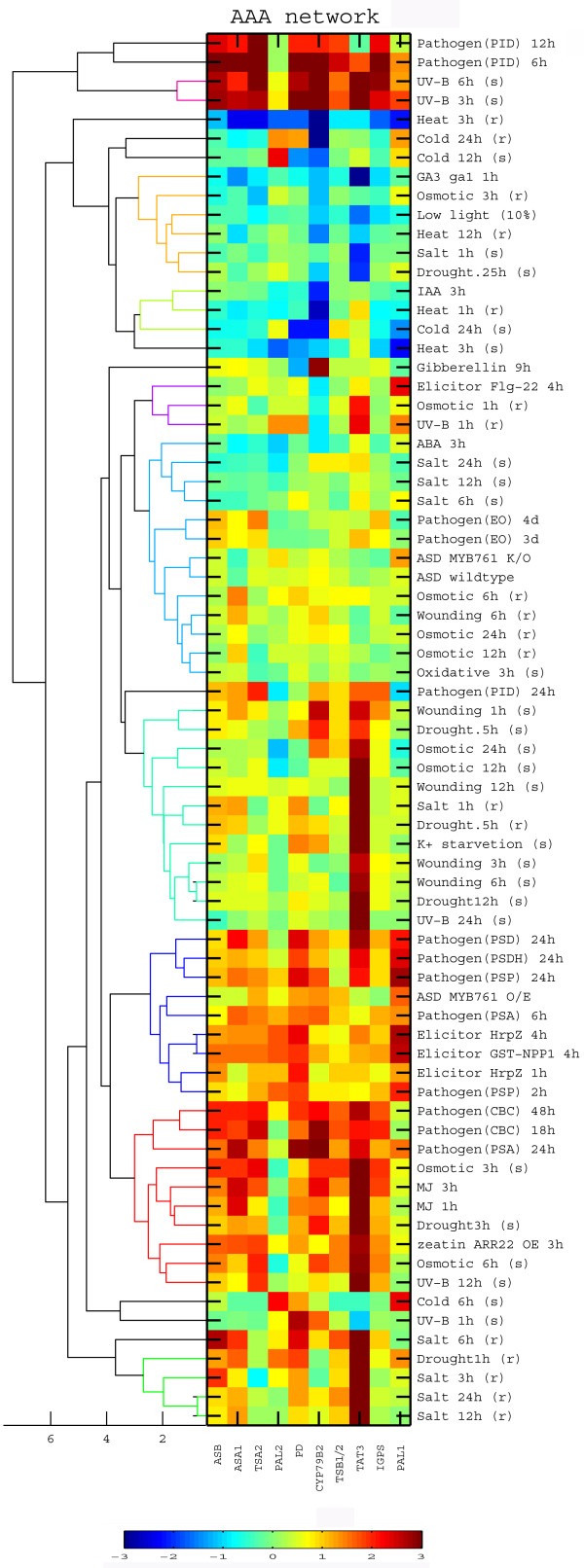
**Clustering of biological perturbations based on their effects of the highly coordinated genes of the Asp-family and aromatic amino acid metabolic networks**. Clustering of the entire set of biological perturbations having a significant effect on at least one of the highly coordinated genes (HCGs) of either the aromatic amino acids or the Asp-family networks based on their effects on the expression of the aromatic amino acids network HCGs. The dendrogram on the left shows the Euclidian distance tree, while the heat map in the center shows the response of the different HCGs to the different biological perturbations that are indicated on the right. The color scale bar is indicated on the bottom. Abbreviations: ASD, adding sucrose in the dark; (s), shoot; (r), root; PSD, CBC, Conidiospores of *Botrytis cinerea*; EO, *Erysiphe orontii*; PID, *Phytophthora infestans *drops; PSA, *Pseudomonas syringae *pv tomato avrRpm1; PSD, *Pseudomonas syringae *pv tomato DC3000; PSDH, *Pseudomonas syringae *pv tomato DC3000 hrcC-; PSP, *Pseudomonas syringae *pv phaseolicola.

Next, we identified the patterns of response of the HCGs of the AAA network to the 211 short-term biological perturbations of the NASC database in an identical manner to that used for the Asp-family network (see above). Fig. 5 lists the specific biological perturbations (right part), as well as their effect on the expression of each of the HCGs of the AAA network (central matrix) and the Euclidian distance between the specific biological perturbations (dendogram on the left). Also in the case of the AAA network, most of the biological perturbations influencing the expression of AAA genes included biotic and abiotic stresses (Fig. 5). The magnitude of the expression responses of the different HCGs to the different abiotic and biotic stresses varied between the different stresses and the time course of each stress (Fig. 5). Interestingly, as opposed to the Asp-family network (Fig. 4), the artificial biological perturbation of adding sucrose in the dark was inseparable from other stresses in its effect on the AAA network (Fig. 5), implying that the hypothetical override of sucrose over dark is network dependent.

### Interactive coordination of expression of the HCGs from the Asp-family and AAA metabolic networks

**Figure 6:**
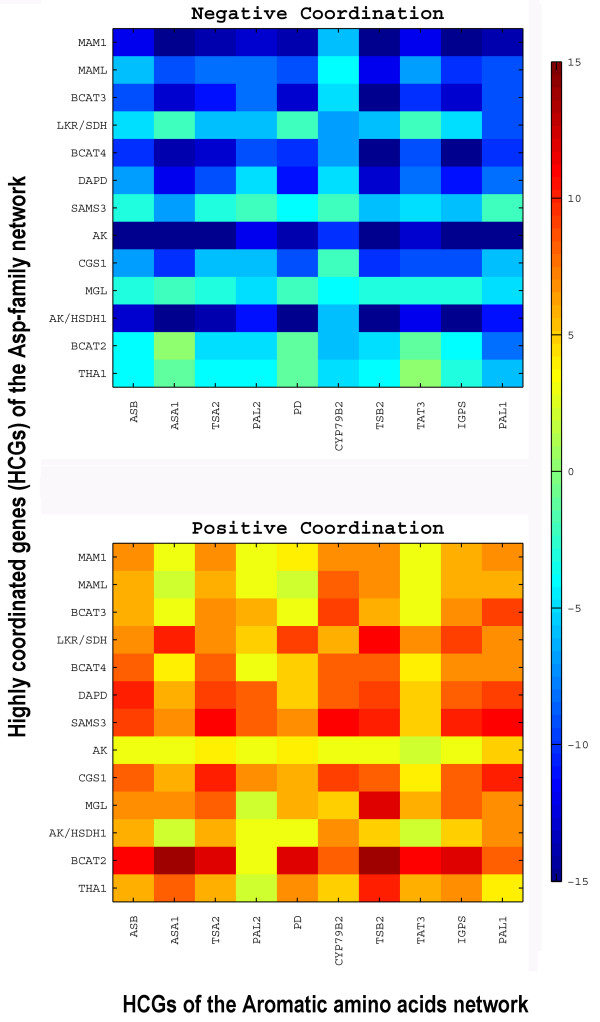
**Negative and positive coordination patterns between the highly coordinated genes of the Asp-family and aromatic amino acid metabolic networks**. Negative (panel A) and positive (panel B) coordination matrixes between the highly coordinated genes (HCGs) of the Asp-family network (Y axis) and the HCGs of the aromatic amino acids (AAA) network (X axis). Color scale bar in indicated on the right.

Next, we examined the cross-network coordination between the HCGs of the Asp-family and AAA networks, based on the same 211 short-term biological perturbations. The respective negative and positive coordination matrixes between the HCGs of the Asp-family and AAA networks are shown in Fig. 6. Notably, most of the HCGs of the two networks exhibited both a considerable cross-network negative coordination (panel A) as well as a considerable cross-network positive coordination (panel B) between them under different biological perturbations. This implies that some cues trigger a coordinated opposite transcriptional regulation of the two networks (or specific branches within these networks), while other cues trigger a coordinated similar transcriptional stimulation or repression of the two networks (or specific branches within these networks). It is important to notice that this behavior, of both negative and positive coordination patterns, observed between the HCGs of both networks, is conceptually different from the behavior that was observed within the HCGs of each network where all pairs of genes were either positively or negatively coordinated and therefore were respectfully assigned to the same or to different groups.

### Interactive influence of different biological perturbations on the Asp-family and AAA metabolic networks

**Figure 7:**
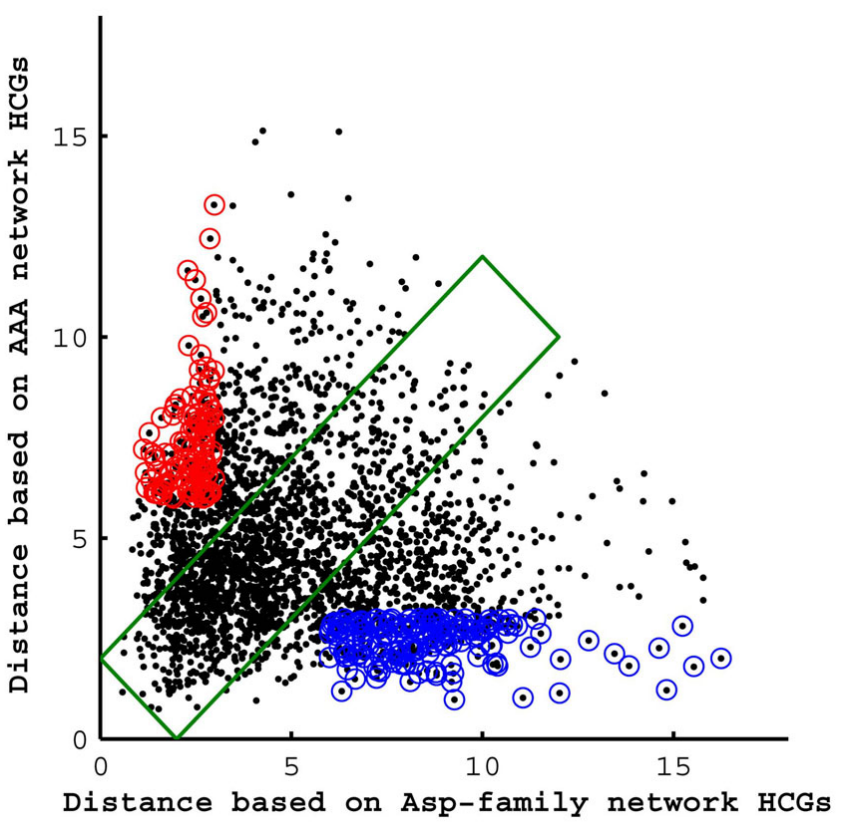
**Classification of the biological perturbations based on their effects on the highly coordinated genes of the Asp-family and aromatic amino acid metabolic networks**. Euclidian distance between pairs of biological perturbations that had a significant effect on one of the highly coordinated genes (HCGs) of either the Asp-family network or the aromatic amino acids (AAA) network. Each black dot represents one pair of biological perturbations in which the position along the X and Y axes represents the differential effect (measured as Euclidian distance) of the pair of biological perturbations on the expression level of the HCGs of the Asp-family network and the AAA network respectively. Black dots inside blue and red circles indicate pairs of biological perturbations having similar effects on the HCGs of one network and differential effects on the HCGs of the second network. Pairs of biological perturbations having the same magnitude of differential effects on both networks (either small or significant) are enclosed in the green rectangle.

Taking into account the central importance of the Asp-family and AAA metabolic networks in plant growth, it was also interesting to examine the extent of interactive effects of different cues (biological perturbations) on the operation of the Asp-family and AAA networks. To address this, we plotted the calculated Euclidian distance between all possible pairs of cues, which significantly alter the expression of at list one of the HCGs in either networks (the same set of biological perturbations that was used for the analyses presented in Figs. 4 and 5), in respect to the magnitude of their differential effects on the entire HCGs of each of the networks (Fig. 7). The pairs of cues (black dots) ranged from pairs having highly similar magnitudes of differential effects (Fig. 7, black dots within a green rectangle) to pairs having progressively increasing differential magnitudes of effects on one of the network while having progressively decreasing differential magnitudes of effects on the second network (black dots outside the green rectangle). We were particularly interested in the black dots within the blue and red circles (pairs of cues having Euclidian distance of > 6 in one network and < 3 in the second network, each representing the extreme ~5% of the perturbations pairs) because they represent pairs of cues having a similar effect on one of the networks, while a differential effect on the second network. These in fact signify cues that are differentially recognized by the plant in respect to the operation of the Asp-family and AAA metabolic networks (see Additional file 2). The entire set of biological perturbations pairs which meet the above criteria can be divided in to two groups: (i) biological perturbations pairs in which both biological perturbations affect one of the networks, while only one of them affects the other network: and (ii) biological perturbations pairs in which both biological perturbations affect both networks, but having similar effects on one of the networks, while differential effects on the other network.

### Identification of genome-wide genes whose expression is coordinated with the expression of the HCGs of the Asp-family and AAA networks in response to the different cues

We also used the gene coordination principle to identify cross-interactions between the HCGs of the Asp-family and AAA networks and other functionally annotated genome-wide genes in order to elucidate the association between the different HCGs groups and specific biological processes. To address this, we selected only genome-wide genes that are either positively or negatively coordinated with all of the genes within each one of the three groups of HCGs (Met metabolism and catabolic group of the Asp-family network and AAA group of the AAA network). This created six groups of genome-wide genes as follows: (i) 920 and 1780 genome-wide genes whose expression is positively and negatively coordinated, respectively, with the Met metabolism group: (ii) 536 and 2451 genome-wide genes whose expression is positively and negatively coordinated, respectively, with the catabolic group; and (iii) 498 and 2466 genome-wide genes whose expression is positively and negatively coordinated, respectively, with the AAA group. The entire lists of these genes are shown in Additional file 3. These genome-wide groups of genes were then classified, using the publicly available gene ontology (GO) annotation  into distinct groups based on their link to biological processes, searching for significant enrichments of specific biological processes that are associated with the different HCGs groups. The entire list of biological processes showing a significant enrichment for each one of the six groups of genome-wide genes is presented in table 2. In general, the Met metabolism group of the Asp-family network was positively coordinated with genes controlling growth-promoting processes, such as nucleosome assembly ribosome biogenesis, translation and biosynthetic processes, while it was negatively coordinated mostly with stress-associated processes, such as the stress-associated hormones jasmonic acid, salicylic acid and ABA as well as trehalose metabolism. The catabolic group of the Asp-family pathway was positively coordinated with various stress-associated process, which only partially overlap with the stress-associated processes that are negatively coordinated with the Met metabolism group of HCGs of this network. In addition, the catabolic group was negatively coordinated with various growth promoting processes, which was only partially overlap with the growth promoting processes positively coordinated with the Met metabolism group of the same network (Table 2). Hence, the Met metabolism and the catabolic groups of the Asp-family network partially negatively interact with each other in respect to their cooperation with other genome-wide genes associated with various physiological processes.

**Table 2:** A list and additional relevant information of all the gene ontology terms that present a significant enrichment in one of the six groups of genome-wide gene coordinated to one of three groups of highly coordinated genes (HCGs).

Coordination	Enrichment (Log_2_)	# of genes in a group	# of genes in the genome	Biological process (GO)
**Asp-family Met met. Group**				
Positive (920 genes)	3.04	4	10	syncytium formation
	2.88	5	14	protein targeting to mitochondrion
	2.86	6	17	microtubule-based process
	2.77	31	94	ribosome biogenesis and assembly
	2.69	5	16	cellular protein metabolic process
	2.67	4	13	nucleocytoplasmic transport
	2.56	12	42	chromosome organization and biogenesis
	2.47	91	340	translation
	2.44	5	19	response to UV
	2.42	7	27	translational elongation
	2.31	13	54	nucleosome assembly
	2.23	5	22	sterol biosynthetic process
	1.73	9	56	fatty acid biosynthetic process
	1.44	17	129	transmembrane receptor protein tyrosine kinase signaling pathway
	-1.62	12	762	regulation of transcription, DNA-dependent
Negative (1780 genes)	2.76	7	11	heat acclimation
	2.66	16	27	response to hydrogen peroxide
	2.57	5	9	response to water
	2.51	15	28	response to high light intensity
	2.41	9	18	cold acclimation
	2.26	9	20	trehalose biosynthetic process
	2.24	8	18	response to desiccation
	2.05	19	49	response to osmotic stress
	2.03	33	86	response to heat
	1.98	36	97	response to water deprivation
	1.9	55	157	response to abscisic acid stimulus
	1.89	8	23	fatty acid beta-oxidation
	1.87	25	73	response to wounding
	1.86	16	47	response to cadmium ion
	1.79	11	34	abscisic acid mediated signaling
	1.76	14	44	toxin catabolic process
	1.68	47	156	response to salt stress
	1.57	25	90	response to ethylene stimulus
	1.47	36	139	response to cold
	1.34	19	80	response to stress
	1.29	23	100	response to jasmonic acid stimulus
	1.26	20	89	response to salicylic acid stimulus
**Asp-family Catabolic group**				
Positive (536 genes)	3.21	6	23	fatty acid beta-oxidation
	2.75	4	21	tryptophan biosynthetic process
	2.71	5	27	response to hydrogen peroxide
	2.66	13	73	response to wounding
	2.27	6	44	toxin catabolic process
	2.27	6	44	aging
	2.15	6	48	defense response to fungus
	2.13	12	97	response to water deprivation
	2.1	19	157	response to abscisic acid stimulus
	1.92	13	122	multicellular organismal development
	1.89	9	86	response to heat
	1.77	15	156	response to salt stress
	1.62	13	150	response to oxidative stress
	1.61	12	139	response to cold
Negative (2451 genes)	2.69	5	6	purine nucleotide biosynthetic process
	2.54	6	8	pentose-phosphate shunt, non-oxidative branch
	2.44	66	94	ribosome biogenesis and assembly
	2.18	199	340	translation
	2.04	9	17	microtubule-based process
	2.01	14	27	translational elongation
	1.95	8	16	cellular protein metabolic process
	1.95	21	42	chromosome organization and biogenesis
	1.89	11	23	tRNA aminoacylation for protein translation
	1.83	11	24	chlorophyll biosynthetic process
	1.72	23	54	nucleosome assembly
	1.32	18	56	fatty acid biosynthetic process
	1.11	36	129	transmembrane receptor protein tyrosine kinase signaling pathway
	1.02	52	199	protein folding
	-0.74	66	851	protein amino acid phosphorylation
	-0.93	36	530	regulation of transcription
	-1.08	22	359	N-terminal protein myristoylation
	-1.19	43	762	regulation of transcription, DNA-dependent
	-1.82	12	329	defense response
	-2.37	5	200	ubiquitin-dependent protein catabolic process
**Aromatic amino acids (AAA) group**				
Positive (498 genes)	4.96	4	5	negative regulation of programmed cell death
	4.14	5	11	phenylpropanoid biosynthetic process
	4.11	4	9	phenylpropanoid metabolic process
	3.47	4	14	aromatic amino acid family biosynthetic process
	3.35	5	19	response to fungus
	3.21	5	21	tryptophan biosynthetic process
	3.14	5	22	cell wall catabolic process
	3	15	73	response to wounding
	2.86	9	48	defense response to fungus
	2.65	5	31	lignin biosynthetic process
	2.37	6	45	defense response to bacterium
	2.14	5	44	toxin catabolic process
	1.86	14	150	response to oxidative stress
	1.74	12	139	response to cold
	1.3	54	851	protein amino acid phosphorylation
Negative (2466 genes)	2.94	6	6	glycine decarboxylation via glycine cleavage system
	2.94	5	5	protein import into chloroplast thylakoid membrane
	2.94	4	4	water transport
	2.94	4	4	photosynthesis, light harvesting
	2.94	4	4	cellulose and pectin-containing primary cell wall biogenesis
	2.94	4	4	amylopectin biosynthetic process
	2.75	7	8	photosystem II assembly
	2.68	5	6	protein import into chloroplast stroma
	2.68	5	6	isopentenyl diphosphate biosynthetic process, mevalonate-independent pathway
	2.68	5	6	carotene biosynthetic process
	2.65	9	11	starch catabolic process
	2.53	6	8	thylakoid membrane organization and biogenesis
	2.48	8	11	reductive pentose-phosphate cycle
	2.36	16	24	chlorophyll biosynthetic process
	1.88	11	23	ATP-dependent proteolysis
	1.77	12	27	chloroplast organization and biogenesis
	1.51	20	54	photosynthesis
	1.31	18	56	fatty acid biosynthetic process
	-0.62	72	851	protein amino acid phosphorylation
	-0.99	50	762	regulation of transcription, DNA-dependent
	-1.22	20	359	N-terminal protein myristoylation
	-1.72	13	329	defense response

The AAA group of HCGs of the AAA network was essentially positively coordinated with genome-wide genes associated with various stresses as well as the production of phenylpropanoids, including the biosynthesis of the cell wall phenylpropanoid lignin, while it was essentially negatively coordinated with various growth-promoting processes, such as water transport, photosynthesis and various biosynthetic processes. Notably, there was a relatively small overlap between the biological processes linked to the Asp-family and AAA networks (Table 2).

## Discussion

### Gene coordination: a new bioinformatics approach to discover genes whose expression pattern exhibits both negative and positive interactions under different biological conditions

Pearson correction is a common bioinformatics approach to search for genes with harmonized expression patterns over a wide range of biological conditions. Yet, in some biological networks, such as networks of amino acid metabolism, some metabolites can serve as substrates to multiple metabolic pathways, leading to the synthesis of different metabolites with differential functions. Due to a potential biological need to channel a given branch point metabolite to different pathways under given growth conditions or to two or more pathways together under other growth conditions, it is expected that expression of given genes encoding enzymes of different pathways may be both positively correlated under some biological perturbations while negatively correlated or not correlated at all under other biological perturbations. In the present report we describe the development of "Gene coordination" as a novel bioinformatics approach to address this issue. This approach takes advantage on the ability to detect positive and negative coordination patterns between gene pairs under multiple biological perturbations by comparing changes in the intensities of expression signals of treatment versus control in several repeated experiments for each biological perturbation. Genes whose expression is either negatively or positively coordinated in a statistical significant manner under a selected set of biological perturbations are defined as highly coordinated genes (HCGs). Using the Asp-family and AAA metabolic networks of amino acid metabolism as model systems, we also provide evidence indicating that HCGs are central regulatory genes within specific biological networks, as evident from their tight negatively or positively coordinated expression with other genome-wide genes under different biological perturbations. Moreover, our results showing high positive coordination with no negative coordination among HCGs of the same group and high negative coordination with no positive coordination between HCGs of different groups within the same network imply that our Gene Coordination approach can identify HCGs groups and networks without any prior experimental knowledge for their existence.

### The Asp-family network is principally regulated by two negatively coordinated transcriptional programs of its HCGs

Our results exposing two negatively coordinated groups of HCGs within the Asp-family network imply that this network is regulated by two opposing transcription programs, namely, when the first program is active the second is repressed and vice versa. The enzymatic steps controlled by the HCGs belonging to these two programs are depicted by blue and red arrows in Additional file 1. The first transcription program (termed Met metabolism program; blue arrows) includes coordinated expression of genes controlling: (i) the entry point into this network (AK); (ii) the conversion of Asp into Lys (DAPD) and Met (CGS); (iii) catabolism of Met via SAM into multiple growth-associated metabolites [3]; and (iv) catabolism Met into multiple glucosinolates (BCAT4, MAM1 and MAML). The second transcription program (termed the catabolic program; red arrows) includes coordinated expression of genes encoding the catabolic enzymes LKR/SDH (catabolizes Lys into Glu and acetyl CoA), THA-1 (catabolizes Thr into Gly) and MGL (Met catabolism into methanethiol as well as to 2-oxobutyrate, an intermediate metabolites of Ile biosynthesis). Each of these two opposing transcription programs also contains a gene encoding a distinct isozyme controlling Ile catabolism into energy production (see Additional file 1; BACT2 in the biosynthesis program and BACT3 in the catabolic program). The biological significance of this observation is still not clear. Notably, even though the Asp-family network also leads to the synthesis of Thr and Ile, genes encoding enzymes of these two branches are not included in the group of HCGs that participate in the transcriptional regulation of this network (see Additional file 1). Yet, the last enzyme of Thr biosynthesis, namely Thr synthase, is regulated by a post-transcriptional control (see [3] and references therein), implying that the principal transcription programs exposed in this report operate in concert with other post-transcriptional programs and covers all branches of the Asp-family network (see next section).

### Concerted transcriptional and post-transcriptional controls of the Asp-family network

Previous studies showed that the Asp-family network is regulated by multiple post-transcriptional controls including: (i) feedback inhibition of the different AK isozymes, controlling the entry point into this network, by Thr, Lys and SAM, feedback inhibition of homoserine dehydrogenase by Thr, feedback inhibition of Thr deaminase by Ile and feedback inhibition of dihydrodipicolinate synthase by Lys (Fig. 1) [3]; (ii) post-transcriptional inhibition of the mRNA level of CGS, the central regulatory enzyme of Met biosynthesis, by SAM via a highly complex post-transcriptional regulation [3]; (iii) stimulation of the activity of Thr synthase, the terminal enzyme of Thr biosynthesis by SAM [3]; and (iv) regulation of LKR/SDH activity by protein phosphorylation [21-23]. Our present results thus add another dimension to the above extensive regulatory programs, implying that the Asp-family network is also regulated by two opposing transcription programs encoding either the "catabolic" or the "Met biosynthesis" groups of HCGs. The participation of the DAPD gene of Lys biosynthesis in the HCGs comprising the biosynthesis program is also particularly interesting, taking into account that a mutation in this enzyme was recently found to play an important regulatory role in the response of Arabidopsis plants to pseudomonas infection, apparently via modulating the regulation of the biotic stress-associated hormone salicylic acid [14,15].

### Interaction of the Asp-family network with genome-wide genes

Our study showed that the Met metabolism and catabolic groups of HCGs of the Asp-family network exhibit: (i) positive coordinated expression patterns with 920 and 536 genome-wide genes, respectively; and (ii) negative coordinated expression patterns with other 1780 and 2451 genome wide genes, respectively (see Additional file 3). Division of these genome-wide genes into functional groups implied the following: (i) the biosynthesis program of the Asp-family network (Met metabolism group) is positively associated with genome-wide growth promoting process (enrichment of genes belonging to growth-associated process including nucleosome assembly, transcription, ribosome assembly and mRNA translation, various biosynthetic processes, microtubule function and intracellular protein targeting), while negatively associated with genome-wide stress-associated process (exposure to various stresses and stress-associated hormones); and (ii) the catabolic program (catabolic group) of the Asp-family pathways is positively associated with genome-wide stress-associated programs (response to various stresses and biosynthesis of stress associated secondary metabolites derived from Trp) and negatively associates with genome-wide growth associated processes (such as nucleotide biosynthesis, ribosome biosynthesis and translation, microtubule function, and biosynthetic processes). Thus, our results suggest that: (i) active growth under favorable growth conditions essentially triggers the biosynthesis of the Asp-family amino acids for their incorporation into proteins and also for the catabolism of Met via SAM towards the synthesis of growth promoting hormones as well for donation of methyl groups for DNA replication and for the synthesis of a large array of growth-associated metabolites; and (ii) exposure to stress conditions stimulates rapid metabolic switches within the Asp-family pathway, shifting fluxes from the Met branch, to the Lys and Thr branches on route towards their catabolism into other metabolites, such as Glu and acetyl CoA (via the LKR/SDH enzyme of Lys catabolism), Ile-mediated energy production (via BACT2) and Gly production (via THA-1), which are apparently needed to support metabolic adaptation to stress conditions. It is also interesting to note that in the Met metabolism group of the Asp-family network, the ratio between the number of genome-wide genes that are significantly positively coordinated to those that were significantly negatively coordinated is much higher than in the catabolic group of the Asp-family network as well as in the AAA group of the AAA network (ratios of 0.52, 0.22 and 0.20 respectively). We hypothesize that this reflects the centrality of the Met metabolism group of the Asp-family network in the physiology of plant growth under favorable, non-stress conditions, an hypothesis that is also in accord with extensive previous literature [3-8]. These observations also support the notion that gene expression networks in plants (like in many other organisms) operate in a modular fashion through interactions of multiple modules, which in the present report are represented by the three HCGs groups of the Asp-family and AAA networks. We also assume that under the majority of growth conditions, most of the modules are relatively inactive, an assumption that is supported by the observation that in most arrays, about half of the genes are not express (data not shown). The outcome of such an assumption is that most modules are expected to be much more often negatively coordinated rather than positively coordinated with other modules, while only central modules, such as the Met metabolism module, which are needed in a wide range of growth conditions, will deviate in respect to their more profound operation.

### The AAA network is principally regulated by a single transcriptional program of its HCGs

Our results imply that the regulation of the AAA network is principally significantly simpler than that of the Asp-family network, including only a single dominant transcription program of 10 HCGs. This transcription program comprises genes controlling the first catabolic steps of Phe, Tyr and Trp and also genes controlling biosynthetic enzymes of Trp (see Additional file 1, enzymatic steps marked with blue arrows). These genes are principally stimulated in response to biotic and abiotic stresses, but the combinations of specific genes within this group of HCGs vary between the different stress conditions. Responses to biotic stresses and also relatively early responses (generally up to 12 hours) to several abiotic stresses, such as UV-B, stimulate the expression of most of the HCGs of the AAA network, while stimulation of only different combinations of the catabolic genes of the different amino acids of this network is principally needed during the late response to various abiotic stresses. As depicted in Table 2, the functions of the genome-wide genes exhibiting positive coordinated expression patterns with the HCGs of the AAA network essentially include: (i) negative regulation of programmed cell death; (ii) facilitation of production of secondary metabolites derived from the aromatic amino acids for various physiological needs, including lignin production for cell wall biosynthesis; and (iii) defence against various biotic and abiotic stresses. The genome-wide genes exhibiting negative coordinated expression patterns with the HCGs of the AAA network generally function in various processes associated with plant growth under favourable growth conditions.

### Regulatory transcriptional interaction between the Asp-family and AAA metabolic networks

The considerable variation in Euclidian distance between many pairs of biological perturbations (mostly stress conditions) when calculated based on their effects on the HCGs of the Asp-family and AAA metabolic networks (Figs. 4, 5 and Additional file 3) has two major implications. The first is that some biological perturbations affect one, but not the second network. For example, both UV-B stress after one hour in shoots and drought stress after one hour in roots stimulate only mildly if at all the HCGs of the Asp-family network (Fig. 4), while stimulating much stronger the HCGs of the AAA network (Fig. 5). Yet, these UV-B and drought stresses seems to differentially affect the AAA network, the first stimulating Phe and Trp catabolism by up regulating PAL3, PD and CYP79B2 and down regulating the TAT3 gene of Tyr catabolism, while the second stimulate only Tyr catabolism by up regulating TAT3 (Fig. 5). The second implication is that some biological perturbations affect both networks, but having different effects on each one of them. For example, UV-B stress after six hours in shoots and low light for 3 h hours in petioles similarly shift the conversion of Met into glucosinolates towards energy biosynthesis by repressing MAM1, MAML and BCAT4 of glucosinolates biosynthesis and up regulating MGL and BCAT2 of Ile-derived energy biosynthesis. Yet these same stresses have differential effect on the AAA network, the first (UV-B after six hours in shoot) highly stimulates Trp synthesis and catabolism (needed to produce sun block metabolites and Trp derived glucosinolate) by up regulation of most of the relevant HCGs, while the second (low light) suppresses the AAA network probably as results of a lower growth rate. An opposite situation is observed in the response to wounding after 12 hours in shoots and the response to salt stress after 12 hours in roots. Both of these stresses promote Tyr catabolism in the AAA network by strongly up regulating TAT3 expression, but having differential effects on the Asp-family network with wounding affecting promoting the catabolism of Thr, Lys and Ile (possibly to enable a short term reduction in Met metabolism), while salt seems to shut down the entire network by down regulating the entire set of biosynthetic HCGs.

## Conclusion

In the present report, we have developed a novel gene coordination approach, which enables the simultaneous identification of positive and negative expression relationships between genes over a wide range of biological perturbations. This approach is particularly important in cases where both positive and negative expression relationships are expected as in genes controlling competing branches in metabolic networks. This approach was central to the identification of HCGs with the Asp-family and AAA metabolic networks of Arabidopsis plants and is likely also to enable the identification of HCGS in other complex biological networks of higher organisms in which only genes encoding a small fraction of the enzymatic steps play a key regulatory role. Notably, our gene coordination approach also revealed that pairs of environmental cues can have a similar effect on one network, while having a differential effect on another network. This implies that when plants are exposed to two or more simultaneous stresses, there may be an override of one stress over the other in its effect on one network, but not the other. Our present report also provides an approach to elucidate such complex relationships.

## Methods

### Data Source and gene expression analysis

Genes encoding enzymes belonging to the Asp-family and AAA metabolic networks were identified and collected using TAIR database  and AraCyc database  supplemented with extensive literature confirmation to avoid false annotations. Gene ontology (GO) annotations were obtained from the TAIR database . Expression data was obtained from the Nottingham Arabidopsis Stock Centre (NASC) , which contains hundreds of publicly available expression profiles. In this study we focused on well documented experiments containing at least two replicate for both treatment and control in which the treatment could be describe as short term response to some external cues. Overall, we selected datasets of 11 different experiments (see Additional file 4). These experiments include 776 microarrays representing 211 different biological perturbations. Gene expression raw data analysis was performed as previously described [19].

### Metabolic network gene coordination calculation & statistical analysis

The first step in the calculation of coordination between each pair of genes was to determine in which biological perturbation each one of the genes has a statistically significant response. In this study we used a standard T-test to calculate the statistical significant of the expression changes in each biological perturbation and used the Benjamini and Hochberg correction procedure to control the false discovery rate (FDR) [24]. The FDR procedure was applied to the group of interest (either genes belonging to the Asp-family or to the AAA metabolic networks) and not to the entire set of genes that were monitored by the microarrays. The out come of this procedure is discrete matrix with 1, 0 and -1 values representing a significant up regulation, no significant change and a significant down regulation, respectively in this matrix which we will name the response matrix each value represent the response of one gene in one biological perturbation. Next, we counted the number of perturbations in which each pair of genes had a similar statistical significant response (both having the value of 1 or -1, positive coordination) and the number of perturbations in which each pair of genes had an opposite, but statistically significant response (one having a value of 1 and the other having a value of -1, negative coordination). Our reason to score significant expression changes with values of 1 or -1 rather than weighting them according to the magnitude of the expression differences, was due to our particular aim to elucidate regulatory coordination maintained over diverse biological conditions that would be masked by weighting according to expression changes. For example, a pair of genes that are significantly coordinated over 10 different biological conditions with a relatively small magnitude of expression change should be, according to our approach, 5-fold more coordinated than a pair of genes that are coordinated over two different biological perturbations with a relatively larger expression change. In order to evaluate the level of significant coordination in each metabolic network, we used the following approach. First, we simulated a response matrix in which there is no coordination between the different genes by randomly swapping the perturbations for each gene separately and recalculating the entire coordination matrix. This process was then repeated 25 times and the positive and negative coordination values were used to estimate the mean and standard deviation of positive and negative coordination for each network. We considered genes as HCGs if they exhibit either negative or positive coordination, which was more then six standard deviation above the background coordination. This stringent value was chosen to ensure that all the selected genes are truly HCGs. The random distributions of positive and negative coordination along with the boundaries values are illustrated in Additional file 5.

### Calculating the distance between biological perturbations

To define the distance between any two biological perturbations, according to either the reaction of the HCGs of the Asp-family or the AAA metabolic networks, we did the following. For each two perturbations we calculated the Euclidian distance using the selected group of HCGs. This analysis with performed using the Matlab function pdist. Since in each biological perturbation the values of the different genes are represented as log ratio of treatment vs. control, no "between studies" normalization was required.

### Identification of genome-wide gene coordination groups

To identify genome wide gene coordination groups for each one of the three groups of HCGs (Met metabolism, Catabolic and AAA groups), we defined that for each gene in each perturbation a P value of less then 0.05 will be considered as a statistically significant response. Next, we calculated positive and negative coordination values between each HCG and the entire set of genes in the genome. Next, we used the background model of coordination, which was calculated separately for each metabolic network, to identify the statistically significant coordination values. Last, we considered a gene to be highly positively or negatively coordinated with a given group of the HCGs if it had a statistically significant positive or negative coordination with all members of that HCGs group.

### Test for gene ontology annotation (GO) enrichment of genome-wide gene coordination groups

Since we were interested in the biological processes that are either positively or negatively coordinated with our HCGs groups, we only tested the enrichment of the biological processes categories in the GO database. For each one of the six groups of genome-wide genes, we counted the number of genes that meet each GO term, and then we applied the Chi2 test to check if this observation is statistically different from the expected number, assuming no enrichment. We only tested terms to which at least four genes were assigned, and we also corrected our P value using the Benjamini and Hochberg correction procedure to control our false discovery rate (FDR). We considered a significant enrichment if the corrected P value was less then 0.05.

## Abbreviations

AAA: Aromatic amino acids; HCGs: Highly coordinated genes.

## Authors' contributions

HL conceived and carried out the study. GG supervised the study and write the manuscript. All authors read and approved the final manuscript.

## Supplementary Material

Additional file 1 **Schematic representation of the Asp-family and aromatic amino acids metabolic networks.** Schematic representation of the Asp-family and aromatic amino acids metabolic networks analyzed in the present report. The positions of the different amino acids in the different networks are marked in boxes. Genes belonging to one of the three groups of highly coordinated genes (HCGs) are marked by color arrows as indicated on the figure, while enzymatic steps whose genes have not yet been identified are indicated by gray arrows. Numbers near each arrow refer to enzyme names as provided in Table 1. (A) The Asp-family network; (B) The Aromatic amino acids network. Dotted lines ending by a bar sign represent feedback inhibition loops. Click here for file

Additional file 2 **Distances between pairs of biological conditions.** Euclidian distances between pairs of biological conditions which are marked in figure 7. Click here for file

Additional file 3 **Probesets belonging to the six groups.** List of probesets and their corresponding genes which are belonging to the six groups of genes used in the enrichment analysis presented in table 2. Click here for file

Additional file 4 **Used datasets.** The list of datasets used to create our combine data set. Click here for file

Additional file 5 **Coordination random distribution.** Distribution of random positive (in red) and negative (in blue) coordination for the Asp-family (panel A) and aromatic amino acids (panel B) metabolic networks created using 25 rounds of simulation. Dash vertical lines represent the threshold values that were selected for each of the metabolic networks. Click here for file
