# Supplementary figures and images for: Coordinations between gene modules control the operation of plant amino acid metabolic networks
Source: BMC Syst Biol. 2009 Jan 26;3:14. doi: 10.1186/1752-0509-3-14 (PMC2646696; doi:10.1186/1752-0509-3-14)

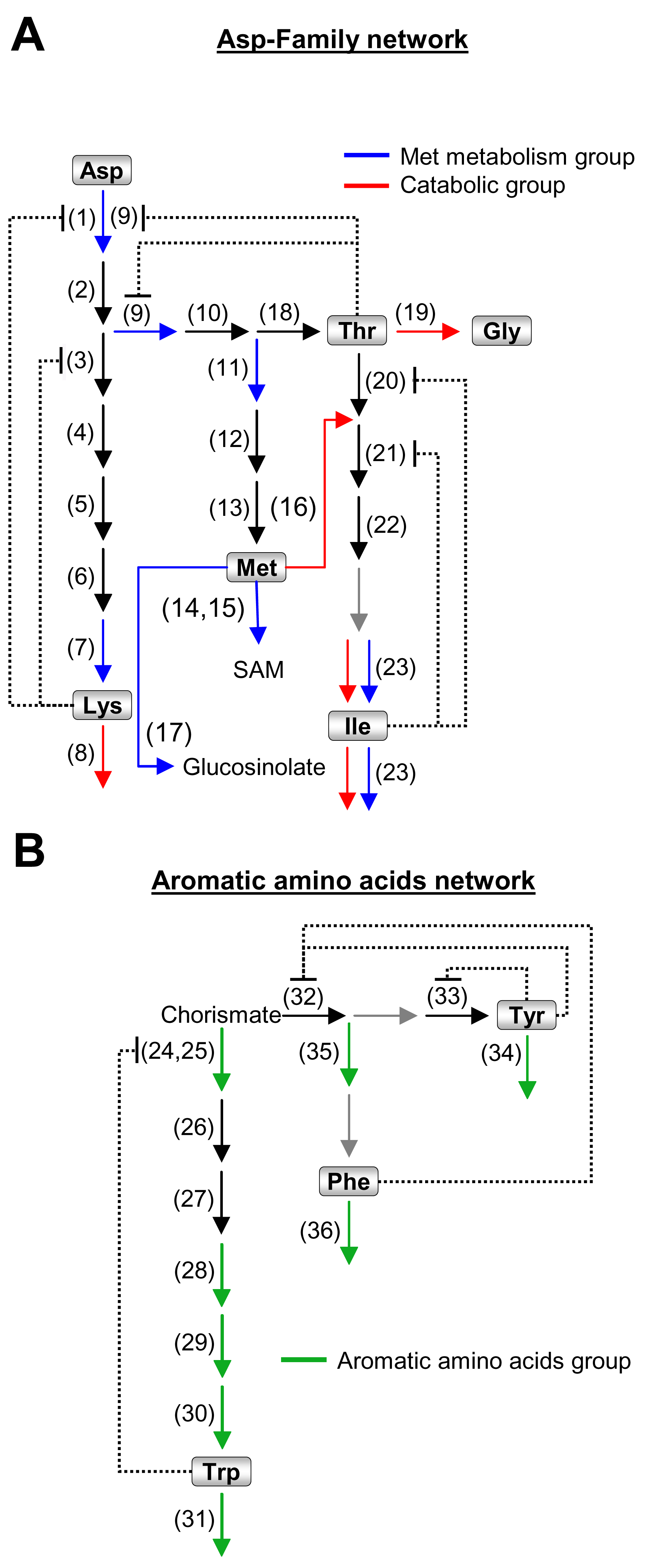

Supplement: Additional file 1 — Schematic representation of the Asp-family and aromatic amino acids metabolic networks. Schematic representation of the Asp-family and aromatic amino acids metabolic networks analyzed in the present report. The positions of the different amino acids in the different networks are marked in boxes. Genes belonging to one of the three groups of highly coordinated genes (HCGs) are marked by color arrows as indicated on the figure, while enzymatic steps whose genes have not yet been identified are indicated by gray arrows. Numbers near each arrow refer to enzyme names as provided in Table 1. (A) The Asp-family network; (B) The Aromatic amino acids network. Dotted lines ending by a bar sign represent feedback inhibition loops. [file 1752-0509-3-14-S1.tiff]

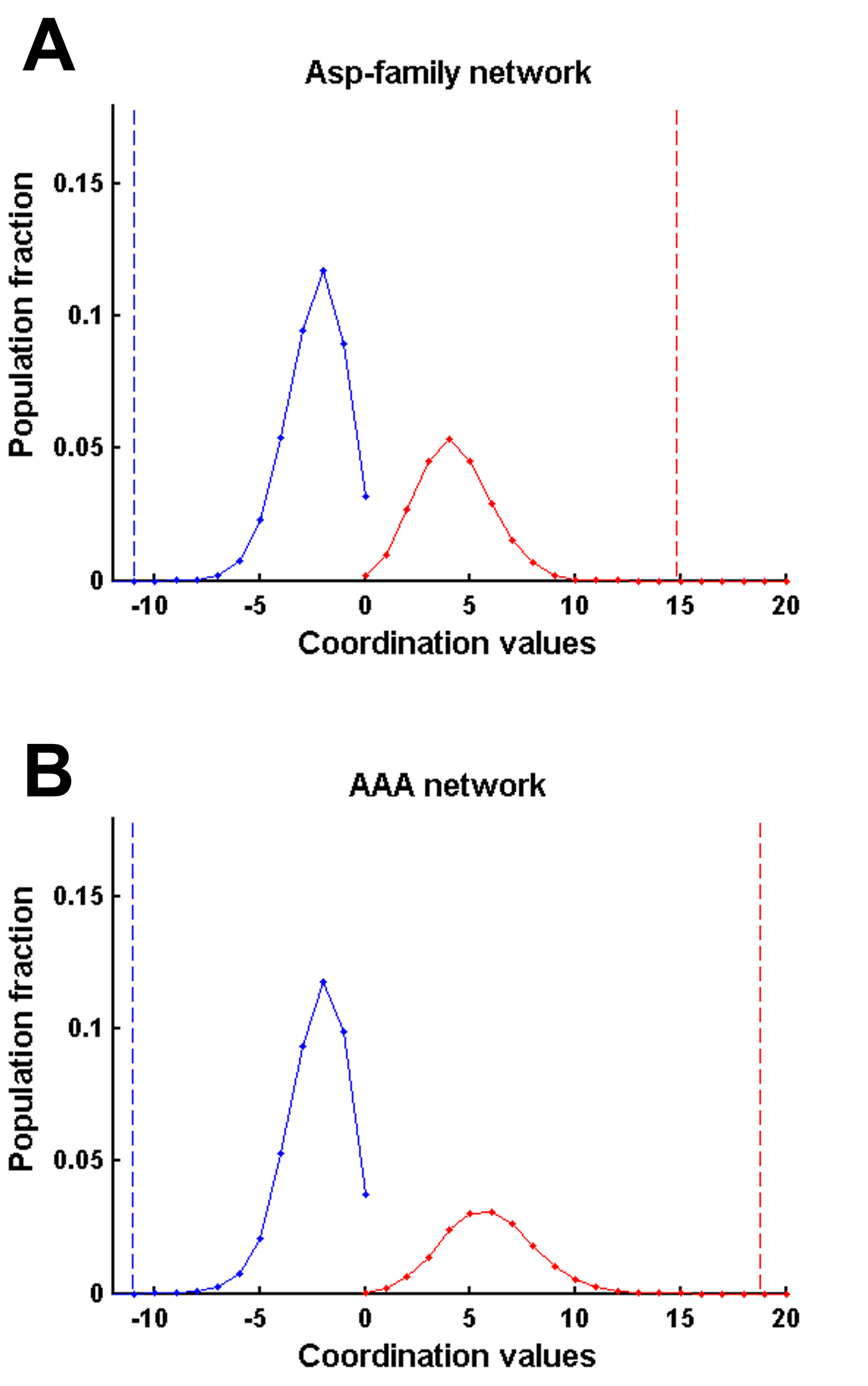

Supplement: Additional file 5 — Coordination random distribution. Distribution of random positive (in red) and negative (in blue) coordination for the Asp-family (panel A) and aromatic amino acids (panel B) metabolic networks created using 25 rounds of simulation. Dash vertical lines represent the threshold values that were selected for each of the metabolic networks. [file 1752-0509-3-14-S5.tiff]
